# Simultaneous determination of twenty-nine active compounds in *fuzhengjiedu granules* by HPLC-QQQ-MS/MS

**DOI:** 10.1016/j.heliyon.2023.e13675

**Published:** 2023-02-11

**Authors:** Yu-Feng Huang, Han-Yue Li, Jun-Xiu Guo, Meng-Xian Wang, Zi-Qi Yang, Xin-Yue Bai, Zhong-De Zhang, Rong-Yuan Yang, Liang Liu, Hua Zhou, Fan He

**Affiliations:** a Guangdong Provincial Hospital of Chinese Medicine, The Second Affiliated Hospital of Guangzhou University of Chinese Medicine, Guangdong, 510006, PR China; b College of Chinese Medicine, Guangzhou University of Chinese Medicine, Guangdong, 510006, PR China

**Keywords:** Fuzhengjiedu granules, COVID-19, HPLC-QQQ-MS/MS, Aconitine, Hesperidin

## Abstract

As an empirical medicine of traditional Chinese medicine, *Fuzhengjiedu Granules* have shown an effect against COVID-19 in clinical and inflammatory animal models. It is formulated with eight herbs, including *Aconiti Lateralis Radix Praeparata*, *Zingiberis Rhizoma*, *Glycyrrhizae Radix Et Rhizoma*, *Lonicerae Japonicae Flos*, *Gleditsiae Spina*, *Fici Radix*, *Pogostemonis Herba*, and *Citri Reticulatae Pericarpium*. This study established a high-performance liquid chromatography-triple quadrupole mass spectrometry (HPLC-QQQ-MS/MS) method by simultaneously determining 29 active compounds in the granules with significant content differences. Separation by gradient elution using acetonitrile and water (0.1% formic acid) as mobile phases was performed on a Waters Acquilty UPLC T3 column (2.1 mm × 100 mm, 1.7 μm). A triple quadrupole mass spectrometer, operating in positive and negative ionization modes, was used for multiple reaction monitoring to detect the 29 compounds. All calibration curves showed good linear regression (r^2^ > 0.998). RSDs of precision, reproducibility, and stability of active compounds were all lower than 5.0%. The recovery rates were 95.4–104.9%, with RSDs< 5.0%. This method was successfully used to analyze the samples, and the results showed that 26 representative active components from 8 herbs were detected in the granules. While aconitine, mesaconitine, and hypaconitine were not detected, indicating that the existing samples were safe. The granules had the maximum and minimum content of hesperidin (27.3 ± 0.375 mg/g) and benzoylaconine (38.2 ± 0.759 ng/g). To conclude, a fast, accurate, sensitive, and reliable HPLC-QQQ-MS/MS method was established, which can simultaneously detect 29 active compounds that have a considerable difference in the content of *Fuzhengjiedu Granules*. This study can be used to control the quality and safety of *Fuzhengjiedu Granules* and provide a basis and guarantee for further experimental research and clinical application.

## Graphical abstract

**Figure:**
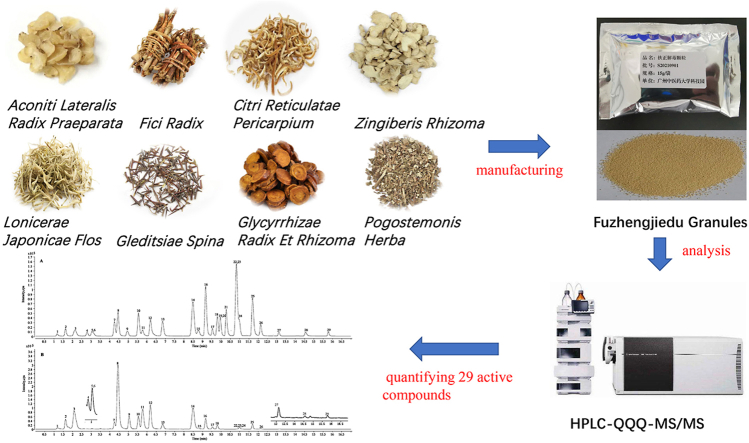

## 1 Introduction

As the virus mutates, the COVID-19 pandemic is still at a global epidemic status, and epidemic prevention and control remain critical. Compound Chinese medicine preparation is a trustworthy option for treating COVID-19, such as Lianhuaqingwen capsules [1,2] and Xuebijing injection [3]. *Fuzhengjiedu Granules* is an empirical formula for clinical adjuvant treatment of COVID-19 [4], especially suitable for severe patients with deficiency syndrome. The previous studies showed that it synergizes in treating COVID-19 [4,5] and animal models of acute lung injury [6], with less herbal toxicity and adverse effects. Therefore, it is an essential and urgent need to establish a multi-component assay to maintain the homogeneity and stability of *Fuzhengjiedu Granules*’ quality, which is also a prerequisite for conducting subsequent clinical applications and mechanism research.

*Fuzhengjiedu Granules* are formulated with eight herbs, including *Aconiti Lateralis Radix Praeparata* (DFP), *Zingiberis Rhizoma* (GJ), *Glycyrrhizae Radix Et Rhizoma* (GC), *Lonicerae Japonicae Flos* (JYH), *Gleditsiae Spina* (ZJC), *Fici Radix* (WZMT), *Pogostemonis Herba* (GHX), and *Citri Reticulatae Pericarpium* (CP). They treat infectious diseases characterized by high fever and cough with phlegm, which could show antibacterial, antiviral, anti-inflammatory, and antipyretic effects. Seven herbs in *Fuzhengjiedu Granules* are included in the Chinese Pharmacopoeia [7], except WZMT [8], which is a local herb delivered from the dried root of *Ficus simplicissima* Lour. In south China and has a similar immune-regulating effect to *Astragali Radix* [9].

It is well known that the most serious obstacle in the natural products development and research is to establish the best quality control method by identifying the representative active chemical constituents in the preparations, especially for complex herbal preparations [10]. The main active components of these eight herbs in *Fuzhengjiedu Granules* are as follows: chlorogenic acid (S**1**), neochlorogenic acid (S**2**), cryptochlorogenic acid (S**3**), caffeic acid (S**5**), isochlorogenic acid C (S**10**), and isochlorogenic acid A (S**12**) in JYH, protocatechuic aldehyde (S**4**) and syringic acid (S**6**) in ZJC, liquiritin (S**7**), isoliquiritin (S**8**), neoisoliquiritin (S**14**), neoliquiritin (S**16**), liquiritigenin (S**18**), isoliquiritigenin (S**24**), and licochalcone A (S**28**) in GC, naringin (S**9**), hesperidin (S**11**), naringenin (S**22**), and hesperetin (S**25**) in CP, benzoylmesaconine (S**13**), benzoylaconine (S**15**), benzoylhypaconine (S**17**), mesaconitine (S**19**), aconitine (S**20**), and hypaconitine (S**21**) in DFP, psoralen (S**23**) and bergapten (S**26**) in WZMT, 6-gingerol (S**27**) in GJ, and pogostone (S**29**) in GHX [7,[11], [12], [13], [14], [15], [16], [17], [18]], respectively. Related literature and studies have shown that these twenty-nine compounds are the foremost active ingredients in each individual. They have significant *in vitro* and *in vivo* pharmacological activities, particularly antiviral, anti-inflammatory, and immunomodulatory effects [19,20]. Therefore, to control the quality and safety of *Fuzhengjiedu Granules*, variations in these twenty-nine compounds can be considered essential quality indicators. However, it is important to note that the levels of these compounds vary. Herbal products contain only pg levels of aconitine and benzoylmesaconine, for example [15,21]. In contrast, hesperidin is as high as at mg level [22]. It is a very challenging task for Compound Chinese medicine analysis to accurately and reliably detect multiple compounds simultaneously.

In the current study, a fast, precise, sensitive, accurate, and concise method by HPLC-QQQ-MS/MS was established to determine twenty-nine significant components simultaneously in *Fuzhengjiedu Granules*. More interestingly, this method allowed simultaneous quantification of different compounds whose levels differ hundreds of thousands of times in the granules. The method can be used to control the quality of *Fuzhengjiedu Granules* products, providing a basis for consistent quality assurance in the mass production of drug manufacturing, clinical application, and experimental research in the future.

## 2 Materials and methods

### 2.1 Chemicals

**Fig. 1:**
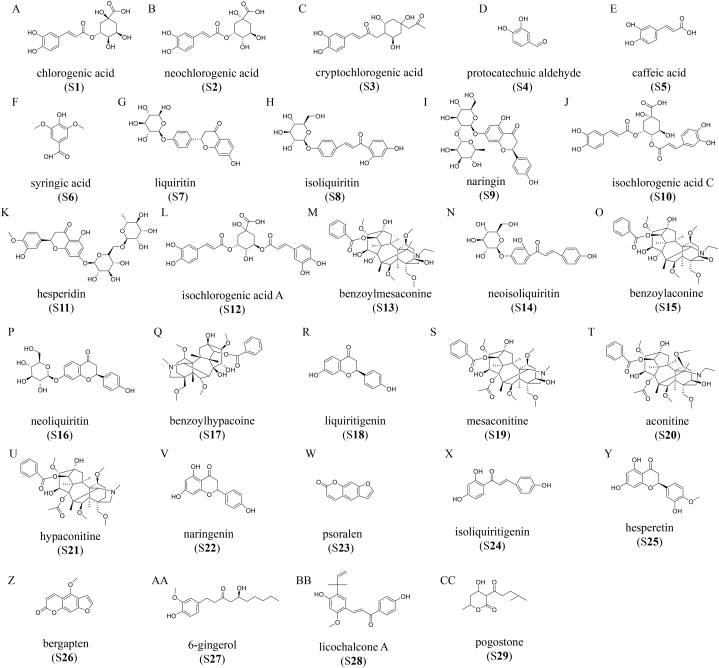
Chemical structures of the twenty-nine active compounds in the *Fuzhengjiedu Granules*: chlorogenic acid (S**1**), neochlorogenic acid (S**2**), cryptochlorogenic acid (S**3**), protocatechuic aldehyde (S**4**), caffeic acid (S**5**), syringic acid (S**6**), liquiritin (S**7**), isoliquiritin (S**8**), naringin (S**9**), isochlorogenic acid C (S**10**), hesperidin (S**11**), isochlorogenic acid A (S**12**), benzoylmesaconine (S**13**), neoisoliquiritin (S**14**), benzoylaconine (S**15**), neoliquiritin (S**16**), benzoylhypaconine (S**17**), liquiritigenin (S**18**), mesaconitine (S**19**), aconitine (S**20**), hypaconitine (S**21**), naringenin (S**22**), psoralen (S**23**), isoliquiritigenin (S**24**), hesperetin (S**25**), bergapten (S**26**), 6-gingerol (S**27**), licochalcone A (S**28**), and pogostone (S**29**) (A-CC).

Reference compounds (purity about 98%, assayed), i.e., chlorogenic acid (S**1**), liquiritin (S**7**), naringin (S**9**), hesperidin (S**11**), benzoylmesaconine (S**13**), benzoylaconine (S**15**), benzoylhypaconine (S**17**), mesaconitine (S**19**), aconitine (S**20**), and hypaconitine (S**21**), and pogostone (S**29**)were obtained from the National Institutes for Food and Drug Control (Beijing, China); neochlorogenic acid (S**2**), cryptochlorogenic acid (S**3**), protocatechuic aldehyde (S**4**), isoliquiritin (S**8**), neoisoliquiritin (S**14**), neoliquiritin (S**16**), liquiritigenin (S**18**), and licochalcone A (S**28**)were purchased from Shanghai Yuanye Bio-Technical Co., Ltd. (Shanghai, China). Caffeic acid (S**5**), syringic acid (S**6**), isochlorogenic acid C (S**10**), isochlorogenic acid A (S**12**), psoralen (S**23**), bergapten (S**26**), and 6-gingerol (S**27**) were purchased from Chengdu Pufei De Biotech Co., Ltd. (Chengdu, China). Naringenin (S**22**), isoliquiritigenin (S**24**), and hesperetin (S**25**) were purchased from Shanghai Macklin Biochemical Technology Co., LTD (Shanghai, China). The chemical structures of twenty-nine compounds are shown in Fig. 1A - Fig. 1CC.

Acetonitrile of HPLC grade was from AQA (Cleveland, USA); Distilled water was further purified by a water purification system (RODI-220A1, RSJ, China); Methanol (Guangzhou, China). The other chemicals were analytical grade. Prior to injection into the HPLC system, all solvents and samples were filtered using 0.22 μm filters.

### 2.2 Apparatus and conditions

Chromatographic analyses were performed on an Agilent 1260 series HPLC system (Agilent Technologies, USA). The system is equipped with a binary pump, an online degasser, an automatic plate sampler, and a thermostat-controlled column compartment. The liquid chromatography condition was performed at 30 °C on a Waters Acquilty UPLC T3 column (2.1mm×100mm, 1.7μm) (Waters, USA). The mobile phase was made up of water (0.1% formic acid) (A) and acetonitrile (B) using a gradient elution of 20–25% B at 0–2 min, 25% B at 2–6 min, 25–45% B at 6–7.5 min, 45–60% B at 7.5–12 min, 60–80% B at 12–13 min, 80–90% B at 13–14 min, 90% B at 14–16 min. The gradient was re-equilibrated for 2 min. The flow rate was 0.25 mL/min, and the sample injection volume was 2 μL.

Mass detection was performed on an Agilent 6460 Triple Quadrupole Mass (Agilent Technologies, USA) equipped with an electrospray ionization (ESI) source. Ionization of the eighteen compounds of S**1**, S**2**, S**3**, S**5**, S**7**, S**8**, S**9**, S**10**, S**11**, S**12**, S**14**, S**16**, S**18**, S**22**, S**24**, S**25**, S**27**, and S**28**, was obtained in the negative ESI mode and the left compounds of S**4**, S**6**, S**13**, S**15**, S**17**, S**19**, S**20**, S**21**, S**23**, S**26**, and S**29** were obtained in positive ESI mode. Direct infusion of each standard solution recorded the MS and MS/MS spectra of the twenty-nine compounds. The multiple reaction monitoring (MRM) transitions were as follow *m/z* 353.10 → 191.10 for S**1**, *m/z* 353.09 → 191.10 for S**2**, *m/z* 353.09 → 173.00 for S**3**, *m/z* 139.04 → 65.10 for S**4**, *m/z* 179.03 → 135.10 for S**5**, *m/z* 199.06 → 140.10 for S**6**, *m/z* 417.10 → 255.10 for S**7**, *m/z* 417.17 → 255.10 for S**8**, *m/z* 579.17 → 271.00 for S**9**, *m/z* 515.22 → 353.10 for S**10**, *m/z* 609.18 → 301.10 for S**11**, *m/z* 515.12 → 353.10 for S**12**, *m/z* 590.30 → 77.10 for S**13**, *m/z* 417.12 → 255.10 for S**14**, *m/z* 604.30 → 77.10 for S**15**, *m/z* 417.12 → 255.10 for S**16**, *m/z* 574.30 → 77.10 for S**17**, *m/z* 255.06 → 119.00 for S**18**, *m/z* 632.30 → 77.10 for S**19**, *m/z* 646.30 → 77.10 for S**20**, *m/z* 616.30 → 77.10 for S**21,** *m/z* 271.06 → 119.10 for S**22**, *m/z* 187.04 → 77.00 for S**23**, *m/z* 255.06 → 119.00 for S**24**, *m/z* 301.07 → 164.00 for S**25**, *m/z* 217.03 → 89.00 for S**26**, *m/z* 293.17 → 57.00 for S**27**, *m/z* 337.14 → 305.00 for S**28**, *m/z* 225.11 → 81.00 for S**29**. Other parameters included: dry gas (N2) flow rate, 11.0 L/min; dry gas temperature, 300 °C; nebulizer, 15 psig; capillary voltage, 4000 V; fragmentor voltage, 80 V for S**6**, 90 V for S**27**, 95 V for S**1**, S**4**, and S**5**, 105 V for S**14**, S**18**, and S**29**, 110 V for S**2**, S**3**, and S**24**, 120 V for S**10**, S**12**, S**22**, S**23**, S**25** and S**26**, 145 V for S**7** and S**16**, 150 V for S**28**, 170 V for S**8** and S**11**, 195 V for S**21**, 200 V for S**17**, 205 V for S**20**, 210 V for S**19**, 215 V for S**9** and S**15**, and 220 V for S**13**; collision energy 14 V for S**1**, S**2**, S**3**, S**5**, S**6**, S**7**, S**8**, S**10**, S**12**, S**14**, S**16**, and S**29**, 18 V for S**24**, S**25**, S**27**, and S**28**, 22 V for S**4**, S**11**, and S**18**, 26 V for S**22**, 30 V for S**9**, 46 V for S**23**, 58 V for S**26**, 114 V for S**15**, 118 V for S**13** and S**17**, 134 V for S**19** and S**20**, 138 V for S**21**.

### 2.3 Standard solutions preparation

**Fig. 2:**
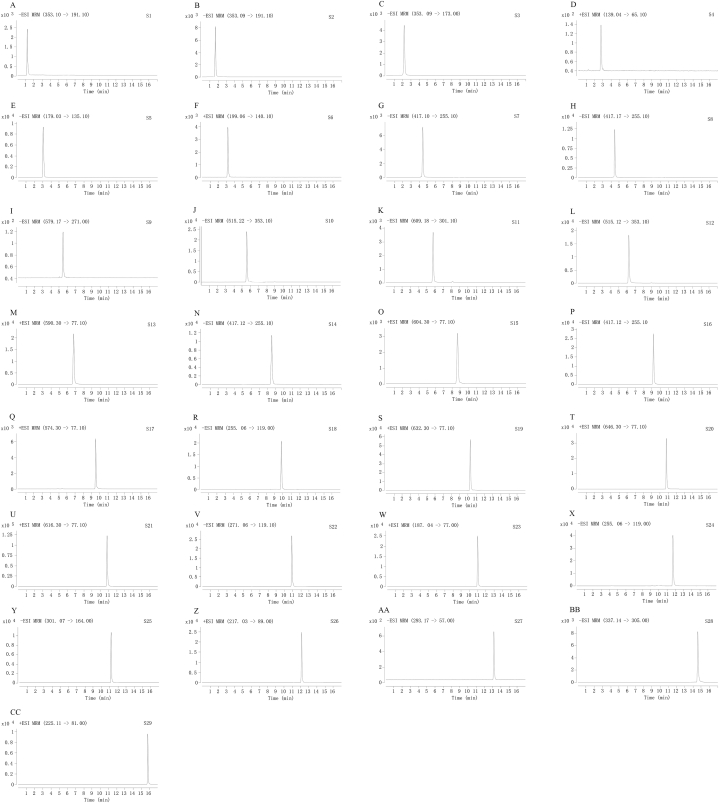
The representative EIC of the twenty-nine standard reference compounds: chlorogenic acid (S**1**), neochlorogenic acid (S**2**), cryptochlorogenic acid (S**3**), protocatechuic aldehyde (S**4**), caffeic acid (S**5**), syringic acid (S**6**), liquiritin (S**7**), isoliquiritin (S**8**), naringin (S**9**), isochlorogenic acid C (S**10**), hesperidin (S**11**), isochlorogenic acid A (S**12**), benzoylmesaconine (S**13**), neoisoliquiritin (S**14**), benzoylaconine (S**15**), neoliquiritin (S**16**), benzoylhypaconine (S**17**), liquiritigenin (S**18**), mesaconitine (S**19**), aconitine (S**20**), hypaconitine (S**21**), naringenin (S**22**), psoralen (S**23**), isoliquiritigenin (S**24**), hesperetin (S**25**), bergapten (S**26**), 6-gingerol (S**27**), licochalcone A (S**28**), and pogostone (S**29**) (A-CC).

Accurately weigh twenty-nine standard compounds and dissolve them with methanol as a standard stock solution. A mixed standard solution was then prepared by accurately mixing the twenty-nine stock solutions with methanol to give concentrations of 0.225 (S**1**), 0.594 (S**2**), 0.516 (S**3**), 0.225 (S**4**), 0.417 (S**5**), 0.284 (S**6**), 2.02 (S**7**), 0.434 (S**8**), 0.618 (S**9**), 0.663 (S**10**), 4.88 (S**11**), 0.50 (S**12**), 0.250 (S**13**), 0.331 (S**14**), 0.376 (S**15**), 0.258 (S**16**), 0.295 (S**17**), 0.193 (S**18**), 0.418 (S**19**), 0.413 (S**20**), 0.626 (S**21**), 0.608 (S**22**), 0.303 (S**23**), 0.541 (S**24**), 0.285 (S**25**), 0.285 (S**26**), 1.06 (S**27**), 0.255 (S**28**), and 0.443 mg/mL (S**29**), respectively. As shown in Table 1, the mixed solution was diluted in order to prepare standard solutions at different ranges of concentration. The calibration curve should be plotted in triplicate with a minimum of six appropriate concentrations. For the analysis, a 2 μL aliquot of the standard solution was injected into the HPLC-QQQ-MS/MS. The representative EICs of the twenty-nine standard compounds are presented in Fig. 2A - Fig. 2CC.

**Table 1:** Calibration curves, LODs, LOQs, precision, repeatability, stability, and recovery of twenty-nine compounds.

Analyte	Linearity			LOD	LOQ	Precision	Repeatability	Stability	Recovery (n = 6)				
	Equation	R^2^	Range (μg/mL)	(ng/ml)	(ng/ml)	RSD (%) (n = 6)	RSD (%) (n = 6)	RSD (%)	Original (μg)	Spiked (μg)	Found (μg)	Recovery (%)	RSD (%)
S1	Y = 18282X+1479.7	0.9982	0.161–2.50	8.06	16.1	1.29	4.44	2.03	0.0141	0.0161	0.0305	101.9	4.02
S2	Y = 1714.1X+9209.5	0.9986	3.48–223	58	174	1.26	2.15	2.09	0.862	0.944	1.83	102.5	1.34
S3	Y = 625.65X +6291.7	0.9980	9.68–155	242	484	0.53	2.02	2.52	0.972	1.06	2.07	103.6	1.02
S4	Y = 772.06X+233.19	0.9988	0.439–14.1	146	439	2.27	3.00	0.33	0.0574	0.0619	0.117	96.3	1.61
S5	Y = 302.94X+149.19	0.9994	1.63–52.1	407	814	4.04	4.07	3.16	0.227	0.244	0.472	100.4	2.53
S6	Y = 219.2X+94.288	0.9995	0.888–14.2	444	888	2.21	3.78	2.63	0.0341	0.0360	0.0688	96.4	2.29
S7	Y = 353.32X+692.62	0.9985	3.16–202	316	631	4.85	4.23	3.82	3.56	3.68	7.32	102.2	1.71
S8	Y = 17373X+122,191	0.9981	6.78–109	3.39	6.78	1.17	1.04	0.58	0.869	0.965	1.79	95.4	1.02
S9	Y = 203.45X+646.12	0.9986	1.93–124	483	966	3.02	2.55	2.44	0.869	0.591	1.44	96.6	1.48
S10	Y = 1073.5X+2567.6	0.9997	2.59–166	129	259	1.46	1.37	2.69	1.95	2.21	4.20	101.8	1.36
S11	Y = 546.34X+34,927	0.9982	30.5–1.95*10^3^	1.53*10^3^	3.05*10^3^	3.40	3.16	2.41	14.2	16.3	30.5	100.0	1.63
S12	Y = 55098X+43,978	0.9987	0.391–25.0	1.95	3.91	0.49	2.41	0.75	0.0597	0.0682	0.126	97.2	1.89
S13	Y = 2292863.8X+66960.9	0.9985	0.0294–0.470	0.0294	0.0588	2.22	4.67	0.63	0.000324	0.000354	0.000693	104.2	3.59
S14	Y = 1912720X+64,006	0.9985	0.0517–0.828	0.0517	0.103	1.01	1.66	0.54	0.00293	0.00320	0.00618	101.6	1.31
S15	Y = 7562629.97X+5341.73	0.9988	0.000977–0.0313	0.0195	0.0326	2.46	4.85	0.62	0.0000200	0.0000217	0.0000418	100.5	2.61
S16	Y = 863.69X+1109.2	0.9990	4.03–258	101	252	4.89	2.00	4.79	2.16	2.25	4.52	104.9	1.61
S17	Y = 6559388.24X+9784.3	0.9982	0.00230–0.0369	0.115	0.289	1.75	4.35	0.27	0.0000472	0.0000499	0.0000990	103.8	2.44
S18	Y = 2088X+1772.9	0.9997	1.51–48.3	37.7	126	2.29	1.59	2.76	0.337	0.362	0.714	104.1	0.67
S19	Y = 4370.6X+203.13	0.9994	0.00816–0.261	2.72	8.16	1.59	–	1.26	–	–	–	–	–
S20	Y = 4397.4X+456.7	0.9983	0.00807–0.516	2.69	8.07	3.10	–	2.55	–	–	–	–	–
S21	Y = 8007X+322.18	0.9985	0.00611–0.196	1.22	3.06	2.13	–	0.88	–	–	–	–	–
S22	Y = 746.59X+228.32	0.9992	0.475–30.4	158	475	1.59	2.09	2.66	0.0208	0.0238	0.0450	101.7	2.33
S23	Y = 1635.2X+2762.3	0.9993	2.37–75.8	118	395	1.00	2.14	0.32	0.365	0.379	0.7439	100.0	1.54
S24	Y = 252.27X+35.541	0.9987	0.338–5.41	169	338	2.58	1.65	4.61	0.470	0.541	1.02	101.7	0.79
S25	Y = 1483.4X+118.93	1.0000	0.111–7.13	55.7	111	4.80	4.47	4.71	0.0172	0.0222	0.0395	100.5	4.86
S26	Y = 1980.6X+2738.5	0.9994	2.23–35.6	37.1	111	1.08	2.07	0.75	0.0786	0.0833	0.161	98.9	1.64
S27	Y = 26.222X+281.8	0.9985	16.5–264	2.06*10^3^	8.25*10^3^	1.89	2.53	2.33	1.76	1.96	3.68	98.0	2.49
S28	Y = 940.78X+144.48	0.9991	0.346–11.1	115	346	4.71	4.39	0.49	0.00329	0.00456	0.00804	104.2	4.47
S29	Y = 694.75X+202.78	0.9990	0.398–25.5	133	398	2.87	4.72	2.23	0.00470	0.00462	0.00927	98.9	4.78

Note: 1. “-” means not determinate.

2. Chlorogenic acid (S**1**), neochlorogenic acid (S**2**), cryptochlorogenic acid (S**3**), protocatechuic aldehyde (S**4**), caffeic acid (S**5**), syringic acid (S**6**), liquiritin (S**7**), isoliquiritin (S**8**), naringin (S**9**), isochlorogenic acid C (S**10**), hesperidin (S**11**), isochlorogenic acid A (S**12**), benzoylmesaconine (S**13**), neoisoliquiritin (S**14**), benzoylaconine (S**15**), neoliquiritin (S**16**), benzoylhypaconine (S**17**), liquiritigenin (S**18**), mesaconitine (S**19**), aconitine (S**20**), hypaconitine (S**21**), naringenin (S**22**), psoralen (S**23**), isoliquiritigenin (S**24**), hesperetin (S**25**), bergapten (S**26**), 6-gingerol (S**27**), licochalcone A (S**28**), pogostone (S**29**).

### 2.4 Sample preparation and analysis

*Fuzhengjiedu Granules* (batch number: S20210901–1, S20210901-2, and S20210901-3) were prepared by Guangzhou University of Chinese Medicine Science and Technology Industrial Park Co., LTD., as following procedures: eight herbal decoction pieces of the prescription were taken, eight times of water was added, and decocted for three times, 1 h each time; The decoction solution was filtered, combined with the filtrate, reduced pressure at 80 °C and concentrated into extract (1 g extract is equivalent to 2 g natural medicine decoction pieces), and the appropriate amount of stevia 0.5% and lactose-dextrin (2:1) were added to make granules and divided into different packaging.

Accurately weighed 1.0 g of *Fuzhengjiedu Granules*, with 20 mL of methanol extracted for half an hour in an ultrasonic water bath (Kunshan Shumei KQ-700 V, China). Before filtration, the weight loss of the extract was compensated with methanol. A syringe filter of 0.22 μm in diameter was then used to filter the extract. As described above, the resulting filtrate was used as a test solution and analyzed by HPLC-QQQ-MS/MS.

## 3 Results

### 3.1 HPLC-MS/MS conditions optimization

**Fig. 3:**
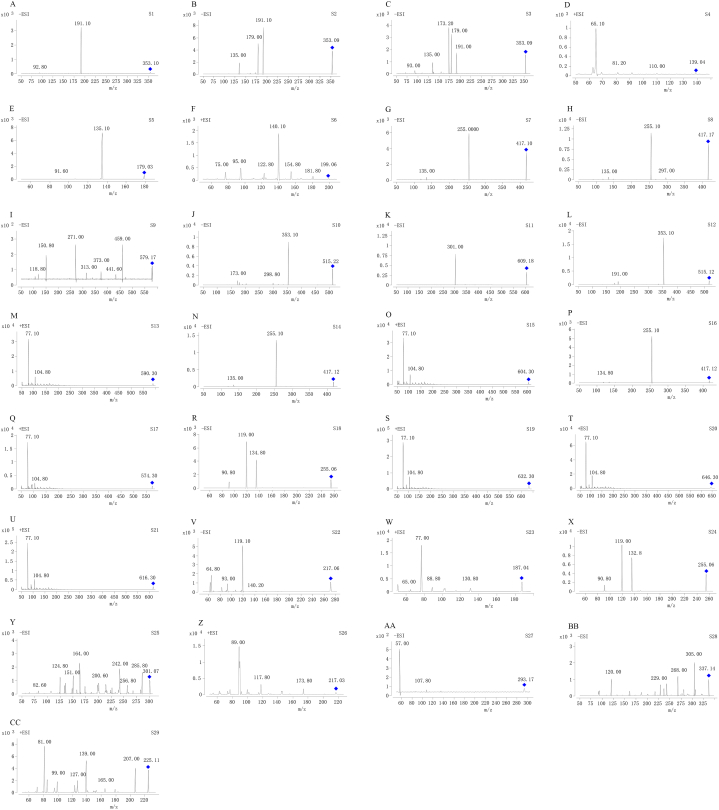
The MS/MS product ion spectra of the twenty-nine compounds: chlorogenic acid (S**1**), neochlorogenic acid (S**2**), cryptochlorogenic acid (S**3**), protocatechuic aldehyde (S**4**), caffeic acid (S**5**), syringic acid (S**6**), liquiritin (S**7**), isoliquiritin (S**8**), naringin (S**9**), isochlorogenic acid C (S**10**), hesperidin (S**11**), isochlorogenic acid A (S**12**), benzoylmesaconine (S**13**), neoisoliquiritin (S**14**), benzoylaconine (S**15**), neoliquiritin (S**16**), benzoylhypaconine (S**17**), liquiritigenin (S**18**), mesaconitine (S**19**), aconitine (S**20**), hypaconitine (S**21**), naringenin (S**22**), psoralen (S**23**), isoliquiritigenin (S**24**), hesperetin (S**25**), bergapten (S**26**), 6-gingerol (S**27**), licochalcone A (S**28**), and pogostone (S**29**) (A-CC).

In order to obtain the best possible resolution and symmetrical peaks of the twenty-nine compounds within a reasonable run time, the chromatographic conditions, in particular the elution conditions of the mobile phase, were optimized. For MS analysis, both positive and negative ion modes were detected. The twenty-nine compounds observed a cleaner background in the mass spectrum and a higher sensitivity in each mode. MRM optimization was performed to obtain the wealthiest relative abundance of parent and product ions by optimizing the parameters of fragmentation voltage and collision energy. The best conditions for HPLC-QQQ-MS/MS are given under the section “2.2. Apparatus and conditions”. Other parameters were set to the values of the inherent parameters of the instrument, such as 11.0 L/min, 300 °C, 15 psig, and 4000 V, including dry gas flow rate, gas temperature, nebulizer, and capillary voltage. Fig. 3A - Fig. 3CC show the twenty-nine compounds' MS/MS product ion spectra.

### 3.2 Method validation

The methodologies were validated by the guidelines for Validation of Quality Standards of Traditional Chinese Medicine (Chinese Pharmacopoeia, 2020, volume 1) [7] and Bioanalytical Method Validation from the US Food and Drug Administration (US Food and Drug Administration, 2018) [23]. Methodological results, including the linear calibration curve with R2, linear range, the lower limit of detection (LOD), the lower limit of quantitation (LOQ), precision, repeatability, stability, and recovery for twenty-nine constituents, are listed in Table 1. All calibration curves reveal good linear regression (r^2^ > 0.998) within the ranges tested. The LODs (S/N ≥ 3) and the LOQs (S/N ≥ 10) of the twenty-nine compounds ranged from 0.0195 ng/mL to 2060 ng/mL and 0.0326 ng/mL to 8250 ng/mL, respectively, indicating high sensitivity. S20210901-1 was continuously analyzed six times on the same day to determine the precision, and the stability test was performed during 0 h, 2 h, 4 h, 6 h, 8 h, and 12 h. Their corresponding relative standard deviations (RSD%) were calculated. Six sample of S20210901-1 were simultaneously extracted and tested as sample preparation. The twenty-nine compounds in the granules were determined by external calibration (standard solution). The RSDs of the precision is less than 5% for all the twenty-nine ingredients (Table 1). In addition, with an RSD of less than 5% for the twenty-nine compounds, the assay also showed a good level of reproducibility. The RSD of twenty-nine compounds within 12 h was less than 5%, which indicated the high level of stability of the sample granules. Recovery tests were performed by adding known numbers of mixed standards to a given amount (0.5 g) of *Fuzhengjiedu Granules*. The test was performed in six replicates. The following equation calculated the recoveries: Recovery (%) = (total amount detected − amount original)/amount spiked × 100%. This novel method is highly accurate, with overall recovery rates of 95.4%–104.9% for the related compounds (Table 1). So far, the HPLC-QQQ-MS/MS method is enough to simultaneously and quantitatively determine the twenty-nine main active ingredients in *Fuzhengjiedu Granules*.

### 3.3 Quantification of twenty-nine compounds in different batches of *fuzhengjiedu granules*

In order to ensure the safety and effectiveness of Chinese herbal products, the determination of bioactive ingredients in Chinese herbal preparations has been established as the most critical concern. However, the quality standards of Chinese herbal preparations only include a few chemical markers, rather than the bioactive compounds of multi-flavor medicinal materials.

**Fig. 4:**
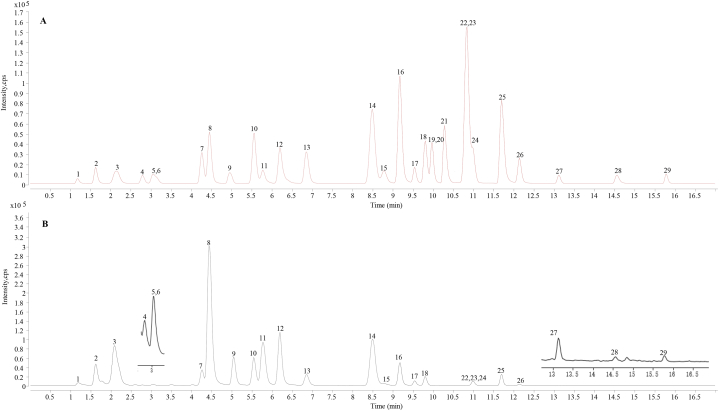
Representative MRM chromatograms of reference compounds mixture (A) and S20210901-1 sample of *Fuzhengjiedu Granules*(B). S**19**, S**20**, and S**21** were not found in the sample solution.

In this study, twenty-nine active compounds in *Fuzhengjiedu Granules* were considered as quality markers of *Fuzhengjiedu Granules*. The twenty-nine compounds are the representative constituents of each herb in *Fuzhengjiedu Granules* [1]. Fig. 4 shows representative MRM chromatograms of reference compounds mixture (Fig. 4A) and endogenous compounds in *Fuzhengjiedu Granules* (Fig. 4B). The twenty-nine compounds were quantitatively determined on the basis of the calibration curves. The levels of the twenty-nine compounds in *Fuzhengjiedu Granules* are shown in Table 2. The level results show the twenty-nine constituents in the three batches of samples (S20210901–1, S20210901-2, and S20210901-3) were in parallel with each other. The content of ingredients between batches is relatively stable in the granules, and the granules are safe and controllable. The content of benzoylmesaconine (S**13**), neoisoliquiritin (S**14**), benzoylaconine (S**15**), benzoylhypaconine (S**17**), licochalcone A (S**28**), and pogostone (S**29**) was relatively low in the granules. While mesaconitine S**19**, aconitine S**20,** and hypaconitine S**21** were even lower than their LOQs. The granules had the maximum and minimum content of hesperidin (27.3 ± 0.375 mg/g) and benzoylaconine (38.2 ± 0.759 ng/g). As *Fuzhengjiedu Granules* is a compound Chinese medicine preparation, it is challenging for the determination and identification of fundamental chemical components. The current method, which can reflect the overall quality of *Fuzhengjiedu Granules*, focuses on quantifying the representative ingredients from each herb. The result performed by this method is highly susceptible accurate, and reliable for analyzing compounds with significant differences in the level of content in *Fuzhengjiedu Granules*.

**Table 2:** The contents of twenty-nine compounds in *Fuzhengjiedu Granules*.

Analyte	Content (mg/g)		
	S20210901-1	S20210901-2	S20210901-3
chlorogenic acid	0.0274	0.0314	0.0286
neochlorogenic acid	1.68	1.68	1.65
cryptochlorogenic acid	1.89	1.92	1.90
protocatechuic aldehyde	0.112	0.111	0.108
caffeic acid	0.443	0.438	0.429
syringic acid	0.0665	0.0647	0.0633
liquiritin	6.94	6.93	7.03
isoliquiritin	1.69	1.70	1.68
naringin	1.69	1.70	1.68
isochlorogenic acid C	3.80	3.89	3.70
hesperidin	27.7	27.5	26.8
isochlorogenic acid A	0.116	0.115	0.111
benzoylmesaconine	6.31*10^−4^	6.33*10^−4^	6.00*10^−4^
neoisoliquiritin	5.71*10^−3^	5.74*10^−3^	5.67*10^−3^
benzoylaconine	3.88*10^−5^	3.86*10^−5^	3.71*10^−5^
neoliquiritin	4.22	4.20	4.11
benzoylhypaconine	9.20*10^−5^	9.35*10^−5^	8.98*10^−5^
liquiritigenin	0.656	0.661	0.654
meaconitine	–	–	–
aconitine	–	–	–
hypaconitine	–	–	–
naringenin	0.0404	0.0416	0.0409
psoralen	0.711	0.714	0.696
isoliquiritigenin	0.915	0.948	0.944
hesperetin	0.0335	0.0344	0.0353
bergapten	0.153	0.154	0.151
6-gingerol	3.43	3.34	3.33
licochalcone A	6.40*10^−3^	7.14*10^−3^	6.09*10^−3^
pogostone	9.15*10^−3^	9.30*10^−3^	8.73*10^−3^

“-” means lower than the LOQs.

## 4 Discussion

*Fuzhengjiedu Granules* is a Chinese herbal compounding with the main effects of invigorating qi and warming yang, eliminating dampness, and detoxifying. It is an empirical prescription for clinical adjuvant treatment of COVID-19, especially suitable for those severe patients with deficiency syndromes. The DFP, ZGC, and GJ originate from Sini Decoction in *Treatise on Febrile Diseases*, which has the function of warming and tonifying the lower yuan; the WZMT tonifies the middle qi; JYH clears away heat and virus; CP, WZMT, and GJ work together to ventilating the lung and resolving phlegm. The combination of various herbs strengthens the body's resistance and detoxifies, invigorates the spleen, and eliminates dampness. The granules' effect was also observed in the clinical practice in treating COVID-19 patients in Wuhan [4], Guangzhou [24], China. *Fuzhengjiedu Granules* can improve the fever symptoms of patients with novel coronavirus pneumonia, promote the absorption of lung inflammation, and reduce the risk of painful death [25].

The twenty-nine compounds from eight herbs in *Fuzhengjiedu Granules* can be divided into four categories: flavonoids, alkaloids, organic acids, and coumarins. Under the Chinese Pharmacopoeia, the selection of potential chemical markers should be based on the following three requirements. The level of the chemical marker in medicinal materials should be greater than 0.02%. Specific or active constituents corresponding to Chinese herbs' function or biological activity should be selected as markers for content determination. Where a single composition cannot represent the overall activity of the drug substance, a multi-component detection method should be adopted [26,27]. Accordingly, the pharmacological activities of S**11** and S**9** from CP, S**7**, S**16**, S**8**, S**24**, and S**18** from GC, S**10**, S**3**, S**2**, and S**5** from JYH, S**27** from GJ, and S**23** from WZMT well represent the major indications or bioactivities of *Fuzhengjiedu Granules* as chemical markers, which are anti-inflammation, anti-virus, antioxidation, immunosuppression. The selected active ingredients in ZJC, GHX, and DFP were meager in granules, especially the content of three diester diterpenoid alkaloids (S19, S20, and S21) was lower than LOQs, indicating a good safety profile for the current batches. S**19**, S**20**, and S**21** are well-known as the toxic ingredients in DFP, which can be hydrolyzed into the less toxic but active ingredients, S**13**, S**15**, and S**17**, respectively, through processing and long-time high-temperature boiling [28]. Therefore, for the safety control of the granules, it is still necessary to take these *Aconitum* alkaloids as safety control indicators. The selection of these compounds as chemical markers was reasonable and representative, which reflected the overall quality of granules based on the multivariate analysis and the bioactivities of these constituents.

## 5 Conclusion

This study developed an efficient and accurate HPLC-QQQ-MS/MS method to quantify twenty-nine active compounds in *Fuzhengjiedu Granules* simultaneously. The method investigated the simultaneous determination of twenty-nine constituents, enormous differences in the content of alkaloids and triterpenoid saponins. The established LC-MS method could be applied to the quantitative evaluation and quality control of *Fuzhengjiedu Granules*. Meanwhile, it could also serve as an exemplary model for the development of quality control methods for chemical preparations of Chinese herbal medicines.

## Author contribution statement

Liang Liu; Hua Zhou: Conceived and designed the experiments; Contributed reagents, materials, analysis tools or data.

Han-Yue Li; Jun-Xiu Guo: Analyzed and interpreted the data; Wrote the paper.

Meng-Xian Wang; Zi-Qi Yang; Xin-Yue Bai: Performed the experiments.

Zhong-De Zhang; Rong-Yuan Yang: Contributed reagents, materials, analysis tools or data.

Yu-Feng Huang: Performed the experiments; Analyzed and interpreted the data; Wrote the paper.

Fan He: Conceived and designed the experiments; Performed the experiments; Analyzed and interpreted the data; Wrote the paper.

## Funding statement

Liang Liu was supported by National 10.13039/501100012165Key Technologies Research and Development Program [2022YFC0867500].

## Data availability statement

Data will be made available on request.

## Declaration of interest's statement

The authors declare that they have no known competing financial interests or personal relationships that could have appeared to influence the work reported in this paper.
